# Zebra Finch chicks recognise parental scent, and retain chemosensory knowledge of their genetic mother, even after egg cross-fostering

**DOI:** 10.1038/s41598-017-13110-y

**Published:** 2017-10-09

**Authors:** Barbara A. Caspers, Julie C. Hagelin, Madeleine Paul, Sandra Bock, Sandra Willeke, E. Tobias Krause

**Affiliations:** 1 Department of Animal Behaviour, Research Group Olfactory Communication, Bielefeld University, Morgenbreede 45, 33615 Bielefeld, Germany; 2 Institute of Arctic Biology, University of Alaska, Fairbanks, Alaska 99775 USA; 3 Institute of Animal Welfare and Animal Husbandry, Friedrich-Loeffler-Institut, Dörnbergstr. 25-27, 29223 Celle, Germany

## Abstract

Mechanisms underlying parent-offspring recognition in birds have fascinated researchers for centuries. Yet, the possibility that chicks recognise parental odour at hatching has been completely overlooked, despite the fact that olfaction is one of the first sensory modalities to develop, and social chemosignals occur in avian taxa. Here we show that Zebra Finch chicks (*Taeniopygia guttata*) are capable of identifying parental odours at hatching. In our first experiment, chicks begged significantly longer in response to the odour of their genetic mother or father compared to the odour of a non-relative of the same sex and reproductive status. In a second experiment, we cross-fostered eggs and tested the response of hatchlings to the scent of genetic vs. foster parents. Chicks from cross-fostered eggs responded significantly more to the odour of their genetic mother than their foster mother, but exhibited no difference in response to genetic vs. foster fathers. This is the first evidence that embryonic altricial birds are capable of acquiring chemosensory knowledge of their parents during early development, and retain chemical familiarity with their genetic mother despite egg cross-fostering. Furthermore our data reveals that kin recognition in birds can develop without any association with a genetic parent at hatching.

## Introduction

Parent-offspring recognition is a fundamental aspect of parental care^1^, and birds are no exception. Studies of many avian species highlight extreme examples of both success and failure to recognize parents and offspring^2–4^. For example, King Penguin chicks (*Aptenodytes patagonicus*) easily recognise parents from a crowd of thousands^5^, whereas Common Cuckoos (*Cuculus canorus*) exploit the lack of recognition of adult Reed Warblers (*Acrocephalus scirpaceus*) that feed enormous cuckoo chicks^6^. Parent-offspring recognition is generally assumed to be based on individual-specific cues, such as vocal recognition. However, altricial chicks do not recognize parental calls, nor do they develop individual-specific calls themselves, until shortly before fledging^3^. Parent-offspring recognition is therefore presumed absent at earlier developmental stages^3^.

Testing the discrimination abilities of chicks at a very early stage provides insight into the underlying mechanisms of parent recognition and potentially kin recognition. The role of chemical perception, in particular, has been notably overlooked in behavioural biology of birds^7,8^. This sensory modality becomes functional in embryos prior to hearing and vision, in a pattern common to other vertebrate taxa^9^. Furthermore, evidence is accumulating that birds have a functioning sense of smell and make use of olfactory cues in social contexts, including sex and species discrimination^10,11^, nest^12,13^, mate^14–16^, and kin recognition^17,18^.

We focused here on olfactory recognition of parental odour during ontogeny in a model altricial songbird, the Zebra Finch (*Taeniopygia guttata*). Chicks of several avian taxa can discriminate between environmental (non-social) odours at an early developmental stage^19–24^. Akin to mammals^25^, olfactory sensory neurons of domestic chicks (*Gallus gallus domesticus*) are also fully functional within the egg six days prior to air-breathing^26^, enabling odour learning *in ovo* ^27^. Recent studies of Zebra Finches provide further evidence of odour learning close to hatching^28^.

**Figure 1:**
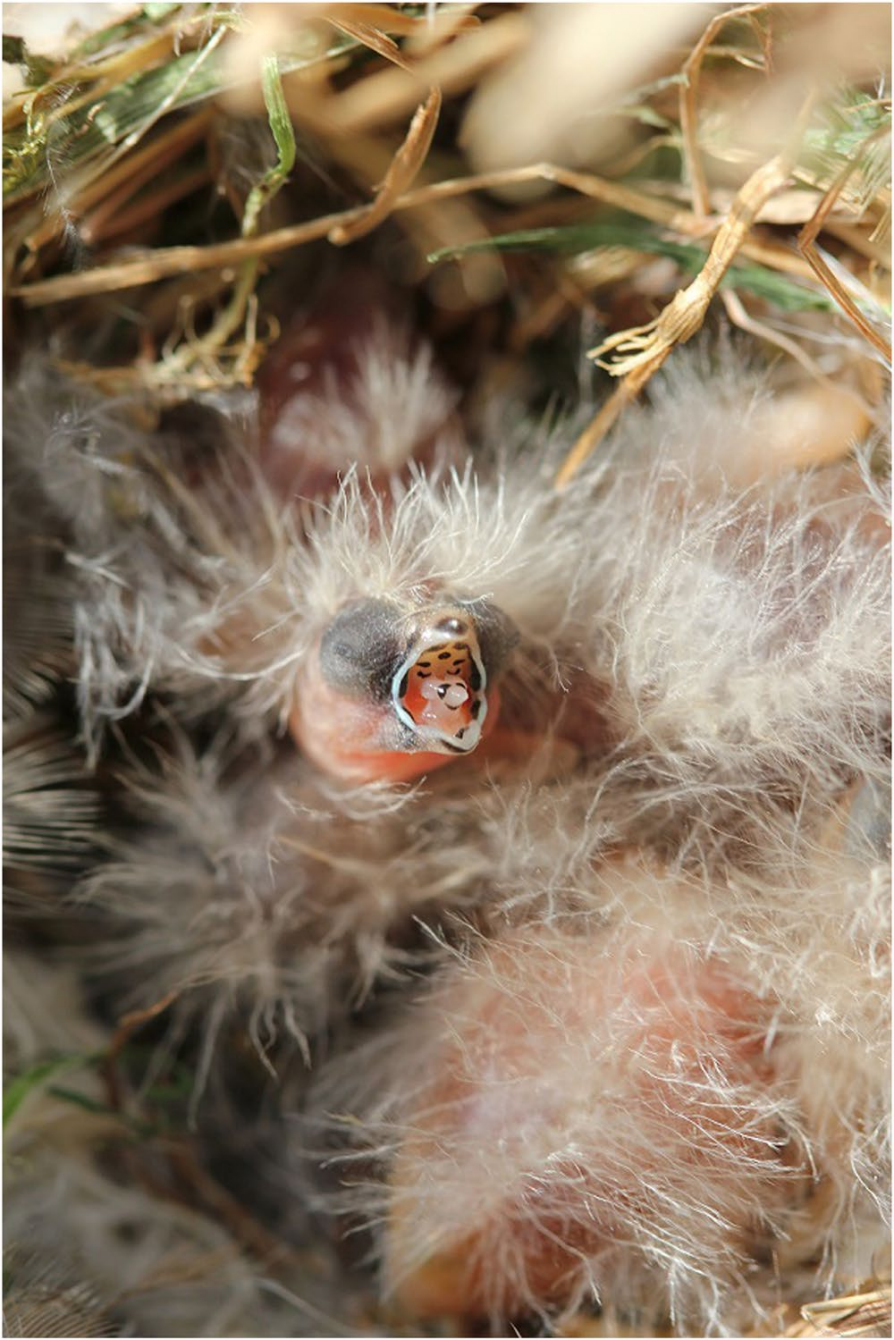
Day-old Zebra Finch chick in its nest, showing stereotypical begging behaviour. The eyes remain closed until about seven days old.

An ability to learn odours early in life led us to ask whether day-old chicks could recognise their parents based on smell alone. Zebra Finches are colonial breeding, monogamous Australian songbirds with biparental care^29^. In addition to acoustic and visual cues^29^, individuals are capable employing olfactory cues during social communication^16,17^. Like other altricial birds, the eyes of Zebra Finch chicks remain closed multiple days post-hatch^29,30^ (Fig. 1), indicating that the sense of smell could be emphasized early in life, in addition to sound and tactile stimulation. Zebra Finch chicks also learn the olfactory signature of their natal nest early in life, potentially in an imprinting process^28^, which enables young to identify their natal nest site after fledging^12^.

In a previous study^23^, we provided proof-of-concept, that day-old chicks discriminated between familiar and unfamiliar artificial scents, as measured by the duration of stereotypical begging. Chicks begged significantly longer towards familiar odours^23^. In the present study, we applied the same begging protocol^23^ to a series of two experiments involving chick recognition of parental odours. The first experiment tested whether chicks were capable of recognizing parental odours under normal nesting conditions. We expected hatchlings would beg longer in response to the familiar odour of each genetic parent, compared to unfamiliar individuals of the same sex, age and reproductive status. The second experiment involved cross-fostering a single egg per clutch into the nest of unrelated adults. If chicks distinguished between the odours of genetic parents vs. foster parents, we expected chick responses would reveal mechanism(s) related to chemosensory learning *in ovo*.

## Results

### Experiment 1: Scent of genetic parent vs. unfamiliar adult

**Figure 2:**
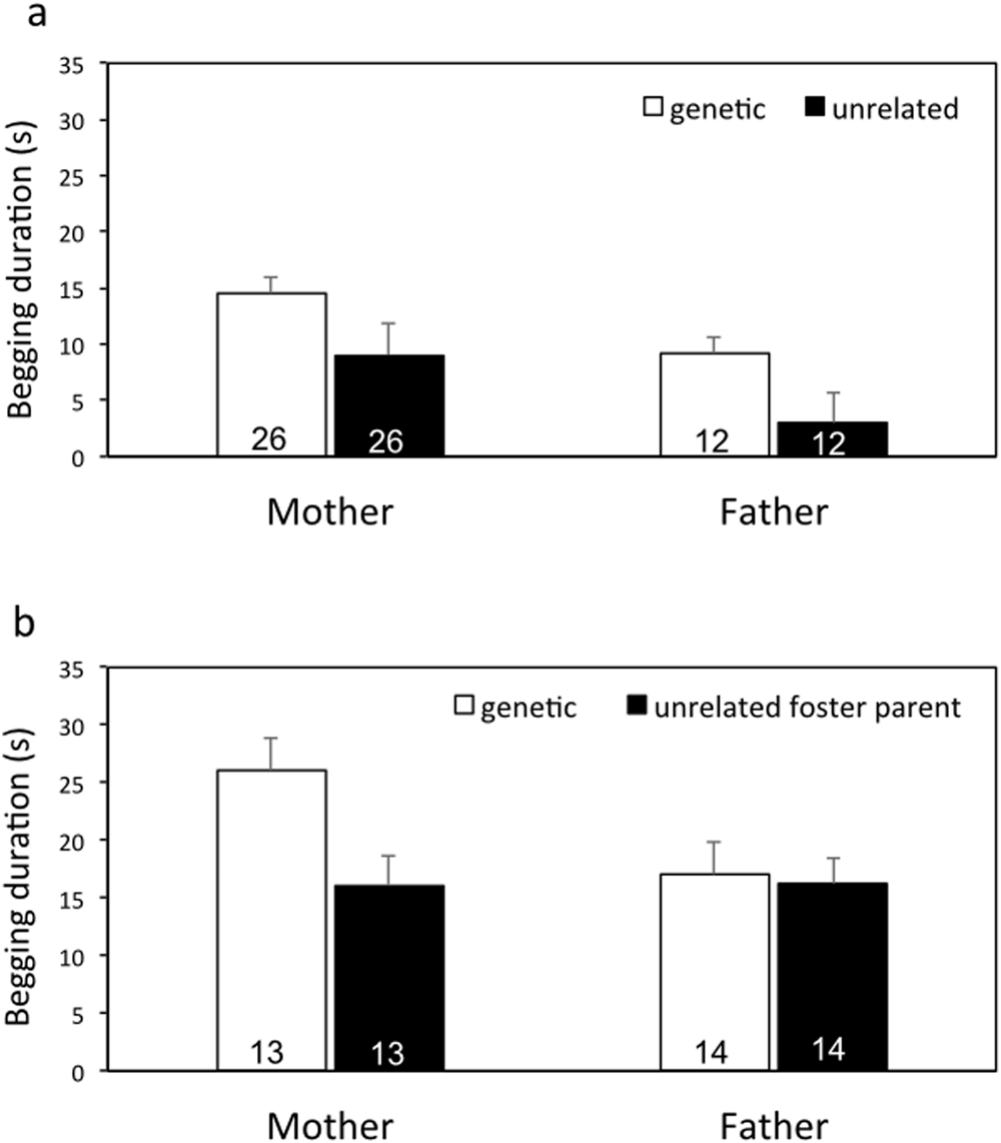
Begging duration (s) of newly hatched chicks in response to two pairs of odour stimuli. Each pair of odors was included the genetic parent and an unrelated adult of the same age and reproductive status tested in randomised order. (**a**) In Experiment 1 (control conditions) chicks revealed a capacity for parent odour recognition by begging significantly longer toward the scent of their genetic mother or father, compared to odor of an unrelated adult. (**b**) In Experiment 2, eggs were experimentally cross-fostered. Foster chicks begged significantly longer in response to the odour of their genetic mothers only, indicating they retained chemosensory knowledge of maternal odour. No such pattern was evident for tests of odor from genetic or foster fathers. Data represent mean + S.E.M. Sample size in each bar represents chicks that exhibited at least one begging response during a two-odour test sequence.

In Experiment 1, we tested 41 chicks from 15 broods to the odour of their genetic mother vs. an unfamiliar female, and 21 chicks from 7 broods to the odour of their genetic father vs. an unfamiliar male under normal breeding conditions. Hatchlings begged significantly longer when exposed to the odour of a genetic parent compared to an unfamiliar adult of the same sex (Fig. 2a, Table 1: Stimulus odour, F_1,36_ = 32.63, p < 0.0001). Chick ID and Nest ID were treated as nested random effects (i.e. chick ID within Nest ID) in our linear mixed model (LME) to account for the non-independence of observations on chicks from the same nest and of paired odour tests (genetic parents vs. unfamiliar adult) on the same chick. The linear model further revealed a significant effect of test type (Table 1: Test type, F_1,19_ = 5.88, p = 0.026), indicating that chicks begged longer during tests of female odour than tests of male odour (Fig. 2a). The meaning of this pattern is unclear, and warrants further investigation. There was no significant interaction between stimulus odour and test type (Table 1).

**Table 1:** Results of LME (Linear Mixed Models) analysis of Zebra Finch begging duration for chicks exposed to natural conditions at a natal nest (Experiment 1) and eggs cross-fostered into nests of non-relatives (Experiment 2).

Factor	df^1^	F	p-value
**Experiment 1 (natural conditions)**			
Stimulus odour (genetic parent vs. unfamiliar adult)	1,36	32.63	<**0.0001**
Test type (adult female or adult male odour)	1,19	5.88	**0.026**
Stimulus odour * Test type	1,36	0.14	0.71
**Experiment 2 (cross-fostering)**			
Stimulus odour (unfamiliar genetic parent vs. foster parent)	1,24	13.02	**0.0014**
Test type (adult female or adult male odour)	1,3	2.15	0.24
Times eggs remained in genetic nest	1,3	2.09	0.24
Stimulus odour * Test type	1,24	10.09	**0.0041**
Stimulus odour * Duration in genetic nest	1,24	0.52	0.48
Test type * Duration in genetic nest	1,3	0.77	0.44

### Experiment 2: Scent of genetic parent vs. foster parent

In the second experiment, we cross-fostered single eggs into the nest of unrelated foster parents. Eggs were transferred after candling confirmed an embryo’s viability, 10 days, on average, after laying (Median: 10; range 6–15). Each egg stayed in the foster nest untill hatching (Median: 3 days, range 1–8). We transferred a total of 38 eggs from 38 different clutches into foster nests, of which 31 chicks hatched. Fifteen foster chicks from 15 broods were presented with the odour of their genetic mother and the odour of their foster mother in a random sequence. The remaining 16 foster chicks from 16 broods were tested to the odours of their genetic father vs. foster father. Begging duration was significantly related to the interaction between stimulus odour and test type (Table 1: Stimulus odour * Test type F_1,24_ = 10.09, p = 0.0041). A post hoc analysis revealed that foster chicks begged significantly longer to the odour of their genetic mothers (Fig. 2b; post-hoc LME p < 0.001), but not to their genetic fathers (Fig. 2b; post-hoc LME p = 0.67). Duration (in days) that eggs had spent in the natal nest prior to cross fostering was not associated with begging duration (Table 1). Other interaction terms were also not significant.

## Discussion

Newly hatched Zebra Finch chicks recognized the odours of their genetic parents (Fig. 2, Table 1), which challenges the long-held assumption that altricial young are unable to discriminate among adults tending the nest^3,31^. Two key results highlight this pattern. First, chicks hatched under natural conditions (Experiment 1) begged longer in response to maternal or paternal body odour (Fig. 2a, Table 1), compared to the scent of an unrelated adult of the same sex. Second, chicks from cross-fostered eggs (Experiment 2) retained an ability to recognise the scent of their genetic mothers only (Fig. 2b, Table 1). Combined, the experiments yield exciting and novel insight into possible chemosensory-based mechanisms underlying early parental recognition in birds.

Developmental sensitivity to chemosensory stimuli *in ovo* raises the exciting possibility that birds imprint to scents they experience early in ontogeny^17,19,21,22^. The chemosenses function in avian embryos prior to hearing and vision^9^ and convey information multiple days before^21,22,26,27^, and just prior to, hatching^19,23,28^. Embryonic chemosensory learning has been described in several vertebrate taxa, including amphibians^32–34^, mammals^25,35,36^ and birds^19,21,22^. Yet, we know relatively little how the process occurs *in ovo* ^19–22^, and even less about the process in altricial songbirds.

The ability of a Zebra Finch chick to recognise the scent of both genetic parents, and in particular, retain knowledge of its genetic mother after cross-fostering, suggests two possible mechanisms: (1) Chemical cues are present within the egg^37^, and/or (2) perceived via gas exchange through pores of the eggshell^38^. Maternal dietary compounds found within the egg, for example, can affect feeding behaviour of newly hatched domestic chicks (*Gallus domesticus*)^37^. Females of many species, including Zebra Finches, can also adjust egg hormone levels^39–41^, suggesting that they may also be capable of providing other types of chemical information during egg formation.

Odour transfer through eggshell has received relatively more attention during bird development^19,21,22,27^. For example, preen gland secretions of female Hoopoes (*Upupa epops*) adhere to eggshell and are likely transmitted through pores to embryos^42,43^. Zebra Finch parents share incubation duties during the day, providing a potential mechanism for exposure to odours of both genetic parents. However, only females develop a brood patch and incubate overnight^44^, during which transmission of maternal odours may be emphasized. Female Zebra Finches can also detect the scent of their own eggs by the end of incubation^45^, suggestive of body odour transfer to eggs. If duration of incubation correlated with the magnitude of parental scent transmission (and opportunity for embryonic chemical learning), then we would have expected eggs spending longer durations in the natal nest to show greater recognition of parental scent. No such pattern was evident (Table 1), but warrants further investigation.

Mechanisms described above, however, do not fully explain chick responses to the scent of genetic fathers. Under natural conditions, birds recognized genetic paternal odour, but cross-fostered chicks did not. Had chemical learning of parental odours occurred primarily during the latter part of incubation, similar to domestic chickens^21^, we would have expected cross-fostered chicks to recognize the scent of their foster parents at hatching. We speculate that Zebra Finch chicks acquire maternal and paternal information differently, similar to studies on visual imprinting^46,47^. Female juvenile Zebra Finches, for example, imprinted to an artificial visually novel trait carried by the father, but not by the mother^47^.

Unlike precocial birds, parental recognition has been assumed absent in altricial species^31^, as there is no obvious necessity. However, a chemically unique scent label^48^, and the capacity to distinguish scent of genetically-related kin (i.e. parents or relatives) has been documented in both European Storm Petrels^18^ (*Hydrobates pelagicus*) and in Zebra Finches^17^. To our knowledge, our data are the first study showing that kin recognition in birds can develop without any association with a genetic parent at hatching. Intraspecific brood parasitism is relatively high in Zebra Finches compared to extra pair paternity^49,50^. Thus maternal cues to assess relatedness may be sufficient in this species. Prenatal information about maternal scent could reduce the risk of maladaptive mating on an offspring’s direct fitness or the mother’s indirect fitness^16^. Our results highlight the impact of parental odours on newly hatched chicks and the potential emphasis of maternally mediated chemosensory information during early ontogeny.

## Methods

### Material and Methods

#### Study organism and breeding conditions

Our experiments were performed from June 2013 - May 2015 at the Department of Animal Behaviour, Bielefeld University, Germany. Zebra Finch males and females used in both experiments represented independent groups of the same domesticated strain^51,52^ (i.e. breeding pairs from Experiment 1 were not the same as Experiment 2). Birds were randomly paired and allowed to breed (one pair per cage) in cages (80 × 30 × 40 cm^3^) with an attached wooden nest box (15 × 15 × 15 cm^3^). The bird room was kept under a 14:10 h light:dark cycle and constant temperature (24.5–25.5 °C). All birds received water and standard seed food *ad libitum* plus additional egg food (Tropical Finches, CéDé, Evergern, Belgium) and germinated seeds three times a week. Coconut fibres were provided as nest material.

#### Chick testing protocol

Nests were checked daily between 0900–1000 hrs for newly hatched chicks. All chicks were tested on the day of hatching during the early afternoon hours (1200–1400 hrs). Each hatchling was exposed to a sequential, two-stimulus odour test. Our test method is described in detail elsewhere^23^, and uses the duration of a chick’s stereotyped begging response as a means of determining odour discrimination.

Briefly, a chick was placed in the experimenter’s palm and exposed to two odour stimuli (described below) in randomized order. The experimenter was blind to the odour sequence and always wore fresh nitrile gloves. Scented air of each odour stimulus was delivered by pressing a wash bottle containing the scent stimulus 10 times in a row (1 press/sec) within 1 cm of the chick’s nares. All odour tests were videotaped and duration of stereotyped begging in response to an odour stimulus was calculated afterward by an observer blind to the odour type and odour sequence. The second stimulus was delivered after the chick had settled from the first odour test. Delivery of both test odours during an experimental sequence lasted approximately 120 seconds total.

In the first experiment, test odours included the scent of a chick’s genetic mother or genetic father, paired with the odour of an unrelated adult bird of the same sex and reproductive status. In the second experiment, cross-fostered chicks were exposed to two different odours consisting of the foster parent and genetic parent, both of the same sex. Each chick was only tested once.

#### Collection of odour stimuli

Odour stimuli were obtained from each parent or unrelated adult Zebra Finch by placing each bird into a nylon sock for 30 minutes^53^. Each adult rested quietly during the odour “collection” period, and showed no signs of stress when released into the aviary afterward (e.g. decreased activity or increased aggression). We used black socks only for all experiments (63% polyamide, 37% cotton, Söckchen Naturelle 60, NUR DIE, DBA Deutschland, GmbH, Rheine, Germany), as different colours of socks emit different volatiles^53^. Following odour transfer, each sock was individually placed into a wide-neck wash bottle (500 ml volume, Rotilabo, Carl Roth). Pairs of odour stimuli were simultaneously prepared just prior to testing each chick^23^. At the end of each test session, bottles and socks were washed with odourless soap (Eubos Wasch + Dusch, parfume-free) and bottles were additionally cleaned with ethanol and dried to remove any residual odours.

#### Experiment 1: Scent of genetic parent vs. unfamiliar adult

Experiment 1 aimed to test whether chicks are able to discriminate between the odour of a genetic parent vs. an unfamiliar adult of the same sex and reproductive status. Zebra finches are biparental and both parents share incubation as well as feeding^29,44^. During the day, males as well as females share incubation, whereas mainly females incubate during the night^44^. As both parents are important for parental care, we expected chicks to recognise the scent of both parents.

In May 2013 we conducted the set of tests involving female odours. We formed 21 pairs of Zebra Finches, which successfully hatched 15 broods and produced a total of 41 chicks. Each chick was presented with the odour of its genetic mother and the odour of an unfamiliar female in randomized order. Next, in October 2013, we tested whether chicks recognized the odour of their genetic fathers compared to an unfamiliar male, by creating another 15 Zebra Finch pairs, which hatched 7 broods and produced 21 chicks for testing. Pairs of odour stimuli originated from the genetic father and an unfamiliar male that was in the same reproductive status.

Both sets of tests (mother and father recognition) were part of two different student projects and therefore performed by different experimenters. We used unique combinations of paired stimulus odours as often as possible (15 pairs of female odour, 7 pairs of male odour). Each chick was tested only once, and individually marked by cutting downy feathers in a unique pattern^54^, before returning it to the nest box.

#### Experiment 2: Scent of genetic parent vs. foster parent

Our second experiment explored the developmental process of parental-odour-learning of chicks, by testing whether eggs cross-fostered and hatched in unfamiliar nests still exhibited odour-preferences for genetic parents. We haphazardly formed 26 breeding pairs in February 2014, of which 15 breeding pairs successfully laid fertile eggs. Nest boxes were checked daily and each egg was marked individually on the day of egg laying by making tiny dots on the shell using a non-toxic pen. Eggs were cross-fostered most often by swapping single eggs of the same age between two clutches. Zebra Finches sometimes bury single eggs from a multi-egg clutch underneath nest material, which results in egg failure. In only one nest, the cross-fostered egg could not be found the next day, which prompted us to cross-foster another egg from the original donor pair.

Each foster brood contained only one egg from another nest and 4 ± 1.5 (mean ± S.D.) eggs from the genetic parents. On the day of hatching, each foster chick was tested to the odour of its genetic mother and the odour or its foster mother, and the odour stimuli were acquired as described above. Whenever a foster chick and a genetic chick hatched simultaneously in the same nest, we performed experiments with both chicks (see Supplementary information) and later determined chick paternity with molecular markers (see below). As in Experiment 1, all chicks were individually marked by uniquely trimming downy feathers^54^.

We repeated a similar set of cross-fostering experiments to test chick responses to odours of foster fathers vs. genetic fathers. In August 2014, 11 of 18 pairs successfully laid eggs, and ten foster chicks hatched for use in odour testing. To increase sample size we re-ran the same tests in February 2015 by creating 20 new breeding pairs (not the same individuals as before), and transferred 12 fertile eggs into foster nests, of which six hatched and were used in odour testing. Chicks hatched from fostered eggs 13.5 days after egg laying (Median 13.5; range 11–15). In total, sixteen chicks hatched and participated in odour testing, as described previously. All behavioural tests in Experiment 2 were performed by the same investigator.

#### Paternity analysis of blood to determine chick identity

We conducted paternity analysis, whenever two eggs in a brood hatched on the same day in Experiment 2, making it impossible to determine identity of the foster chick. A small blood sample (5–10 µL) was taken from both chicks and parents via brachial venepuncture using a 26-gauge needle and stored in 70% ethanol. Total genomic DNA was extracted using an adapted phenol-chloroform protocol. Each sample was then genotyped at 8 polymorphic microsatellite loci^55,56^: Indigo41, Tgu01, Tgu03, Tgu04, Tgu05, Tgu08, Tgu09 and Tgu12 using a Qiagen Type-it Mastermix kit. The following PCR-profile was used: 95 °C for 5 min, 8 cycles of 95 °C for 30 seconds, 60 °C for 90 seconds (minus 1 °C per cycle) and 72 °C for 60 seconds, followed by 30 cycles of 95 °C for 30 seconds, 56 °C for 90 seconds and 72 °C for 60 seconds. Final extension was performed at 70 °C for 15 minutes. Fragment sizes were scored using the automated software Genemarker v1.7 (Softgenetics). All of the scores were checked manually and adjusted wherever necessary. Paternities were assigned manually to one of the two potential parent pairs by comparing the fragment sizes. Paternities were assigned unambiguously in all cases (n = 7 foster chicks; 4 in the experiment with the maternal scent and 3 in the experiment with the paternal scent).

#### Ethical Note

The blood sampling was carried out under the permission (# 84-02.05.20.12.283) of the LANUV NRW. Housing and bird breeding were approved by the veterinary office, Bielefeld, Germany (# 530.421630-1, 18.4.2002). All birds remained in the aviary stock after experimentation. All experiments were performed in accordance with the animal experimentation guidelines and laws of Germany.

#### Data analysis

Experiment 1 aimed to test whether chicks can differentiate between the odour of their parents and the odour of an unfamiliar conspecific. We used a linear mixed model (LME) with begging duration as the response variable. Predictor variables included stimulus odour (familiar related/unfamiliar unrelated), test type (mother recognition/father recognition), and interaction term (test type*stimulus odour). Chick ID and Nest ID were treated as nested random effects (i.e. chick ID within Nest ID) with random intercepts, to account for the non-independence of observations on the same chicks and of chicks of the same nest.

Data from Experiment 2 were also analyzed via LME in the same manner stated above. However, we also included the duration (in days) that eggs remained in the genetic nest prior to cross-fostering in this model. Eggs varied somewhat in age prior to cross-fostering (see Results), and this term tested whether differential exposure to odours of genetic parents was related to chick response. The model also included two-way interactions of predictor variables.

In both Experiment 1 and Experiment 2, any chicks that did not beg toward either scent in a test sequence were excluded from the analysis, as they provided no data relevant to our research question. During Experiment 1 a total of 24 chicks did not beg to both test stimuli (n = 15 during tests of maternal odour, and n = 9 during tests of paternal odour). In Experiment 2 only 4 chicks failed to beg (n = 2 each during tests of maternal odour and tests of paternal odour). For Experiment 2 post hoc analyses were conducted by running a LME separately for the two test types (mother and father, respectively). All models were run in R. 3.3.1 using the nlme package^57^. Validity of each model was confirmed via visual inspection of the residuals for normality (plus Shapiro-Wilk tests, all n.s.) and heteroscedasticity. We considered P values ≤ 0.05 to be statistically significant.

### Data availability statement

All data generated or analysed during this study are included in this published article (and its supplementary information files).

## Electronic supplementary material

Supplementary Information

Dataset 1
