# Supplementary material for: Zebra Finch chicks recognise parental scent, and retain chemosensory knowledge of their genetic mother, even after egg cross-fostering
Source: Sci Rep. 2017 Oct 9;7:12859. doi: 10.1038/s41598-017-13110-y (PMC5634463; doi:10.1038/s41598-017-13110-y)
Supplement: Supplementary file 1 — Supplementary Information [file 41598_2017_13110_MOESM1_ESM.doc]

**Supplementary information**

**
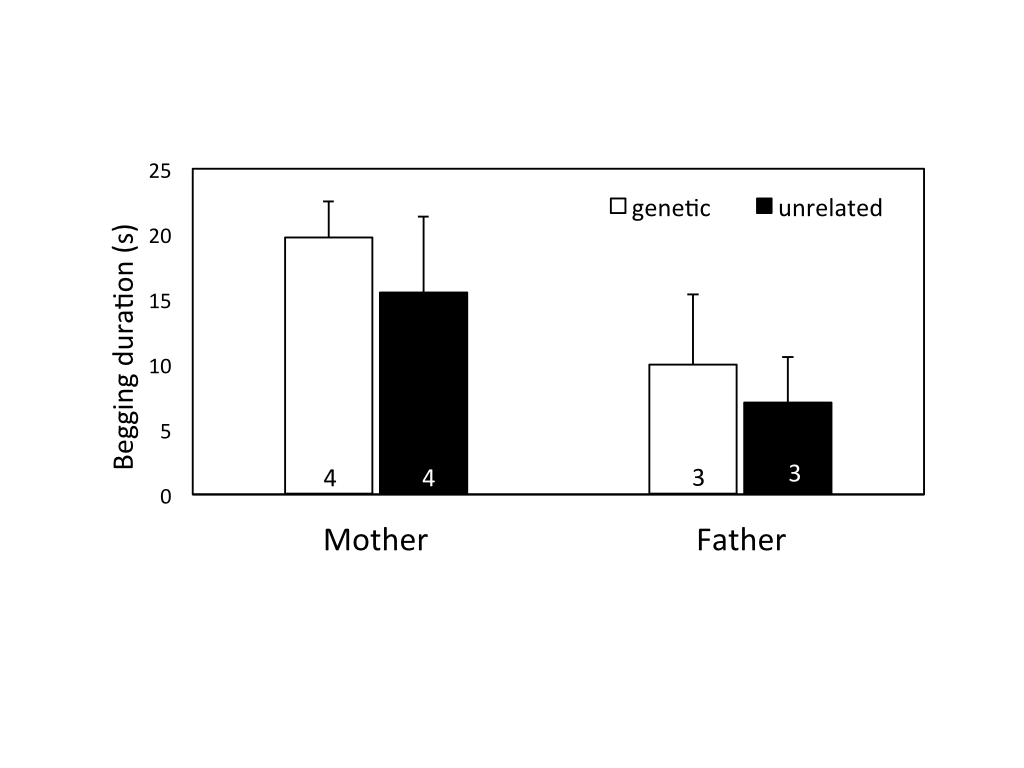
**

**Figure 1S: Results of seven chicks genetically related to incubating parents, but tested during Experiment 2. Each of these chicks hatched simultaneously with the cross-fostered egg (in the same nest), but could not be distinguished prior to odour testing. Subsequent paternity analysis revealed the chicks’ identity. Similar to Experiment 1, (a) Four chicks were tested to the scent of their genetic mother and an unrelated female, and (b) Three chicks were tested to the scent of their genetic father and an unrelated male. Data represent mean + S.E.M.**
